# Parliament and the Profession

**Published:** 1916-08-26

**Authors:** 


### PARLIAMENT AND THE PROFESSION.

### Rubber Gloves for Nurses.

Replying to Major Sir C. Hunter, Mr. Forster said that every demand made by medical officers for rubber gloves for the use of nurses in military hospitals had been met.

### Russian Doctors and Commissions.

It is impossible, said Mr. Forster, in reply to a question, to grant commissions to medical men who are not British subjects and whose degrees are not registerable in this country.

### Wounded Soldiers.

Mr. Jowett asked a question which suggested that the military authorities are acting unfeelingly "by sending back to the trenches men who have newly recovered from wounds, in many instances time after time, before their turn comes to go into the trenches again." Mr. Forster, however, assured the House that men are not sent back to the Front until they have fully recovered from wounds and are fit for service in the field.

### Hairmyres Sanatorium.

The Secretary for Scotland informed Mr. MacCallum Scott that additional workmen had been obtained for the completion of the construction work of the Hairmyres Sanatorium, at which the Middle Ward Committee of the Lanarkshire County Council had pledged itself to afford accommodation for discharged tuberculous soldiers and to train them for outdoor occupation.

### Cocaine.

Illicit traffic in cocaine in India, said Mr. Chamberlain, continues to be a source of grave anxiety, and stringent regulations have been framed. Imports are prohibited except under licence, private possession is punishable, and sale to private persons by chemists is permitted only for medical purposes on the prescription of a qualified medical practitioner. The drug, however, lends itself to smuggling by seamen and others in ways which defeat the best conceived regulations.

### The Health of Munition Workers.

Every effort is made to provide suitable medical and nursing services in munition factories so far as may be necessary. But, said Mr. Long, replying to Mr. Macmaster, it is obvious that the means for obtaining these services are affected by the demands for medical services on the part of the military and naval authorities and of the civil population.

### Women Army Doctors.

Mr. Dickinson asked whether women doctors were now being employed upon foreign service under the War Office, and, if so, whether they were entitled to receive the same pay as male doctors so employed. Mr. Forster replied that women doctors would receive the same pay and allowances as male doctors similarly employed.

### Elham Hospital.

Mr. Bennett-Goldney asked the Secretary for War whether he would take the necessary steps to accelerate the work which had been considered advisable to undertake in connection with the conversion of the Union at Elham into a hospital for sick Canadian soldiers. Mr. Forster, who replied, said the building had been allotted to the Canadian authorities, and they were making their own arrangements.

### Medical Arrangements in Mesopotamia.

A statement respecting the present medical arrangements in Mesopotamia was made by Mr. Lloyd George, in reply to a question by Sir John Jardine as to whether the Secretary of State for War could assure the House that sufficient supplies of drugs, medicines, bandages, and other medical appliances have reached the troops at Basra and in the stations on the lines of the Tigris and the Euphrates. Mr. Lloyd George said that, subject to its being understood that the Army Council did not claim that all had yet been done which should be done and will be done, he might state that on all new boats ordered in the United Kingdom arrangements for securing adequate proper drinking-water had been made. Filtration barges are being sent, and supply tanks on various points in the river will be provided. As regards the vessels already on the spot, an installation for sterilising water, recommended by the Director of Medical Services and the late Sir Victor Horsley, had already been provided on many ships, and will be fitted to the remainder. Two tanks holding 40 gallons for cooling purposes had also been added on each vessel. A large number of hospital river steamers and barges have also been ordered. As regards drugs, medicines, bandages, and other medical appliances, all demands have been supplied, and, added Mr. Lloyd George, "as the General Officer Commanding has been repeatedly told to ask for all he wants, I am satisfied that sufficient supplies are now available on the spot."

### Other Matters of Interest.

Non-poisonous Dope.—Satisfactory dopes which are free from tetrachlorethane are now available in sufficient quantity for use by aeroplane contractors, and as a consequence Mr. Brace was able to state that there has been a considerable decrease recently in the number of cases of jaundice due to dope.

Venereal Diseases.—No complete schemes for the diagnosis and treatment of venereal diseases have as yet been submitted to the Local Government Board by local authorities, but, said Mr. Hayes Fisher, numerous negotiations and conferences have taken place, and considerable activity is being displayed by local authorities in carrying out the duties imposed on them.

